# Supplementary material for: ER stress-linked autophagy stabilizes apoptosis effector PERP and triggers its co-localization with SERCA2b at ER–plasma membrane junctions
Source: Cell Death Discov. 2019 Sep 3;5:132. doi: 10.1038/s41420-019-0212-4 (PMC6718399; doi:10.1038/s41420-019-0212-4)
Supplement: Supplementary file 1 — Supplemental Material File #1 [file 41420_2019_212_MOESM1_ESM.docx]

Supplementary Figure 1. **Halo-PERP plasma membrane localization and functional characterisation.** **(A)** Halo-PERP fusion protein localization was assessed in Mel202 cells using the fluorescent HaloTag TMRDirect ligand. Scale bar 20 μm. **(B)** Protein levels of endogenous PERP and p53 in Mel202 cells expressing HaloTag (24 h) or Halo-PERP for the indicated time points. One-way ANOVA, n=3, p53: F=13.39, p=0.0001***; PERP: F=17.12, p<0.0001****. **(C)** The percentage of apoptotic Mel202 cells expressing HaloTag and Halo-PERP labelled with the R110 HaloTag fluorescent ligand 48 h post-transfection determined by flow cytometry using Alexa Fluor 647 annexin V. Student’s t-test, n=3, p=0.0026**.
